# Determining the impact of a self-care educational program designed based on the Peplau theory on adherence to treatment and self-care in elderly patients with diabetes

**DOI:** 10.17533/udea.iee.v43n1e05

**Published:** 2025-04-21

**Authors:** Matin Roostaye Abkenar, Elham Imani, Saeed Hosseini Teshnizi, Neda Sadat Ahmadi, Yasin Moradi

**Affiliations:** 1 Master's student in medical surgical nursing, student research committee. Email: matinr1997@gmail.com https://orcid.org/0000-0001-9121-0000 Hormozgan ‎University of Medical Sciences student research committee Iran matinr1997@gmail.com; 2 Associate Professor, Mother and Child Welfare Research Center. Email: eimani@hums.ac.ir. Corresponding author. https://orcid.org/0000-0003-3957-3343 Hormozgan ‎University of Medical Sciences Mother and Child Welfare Research Center Iran eimani@hums.ac.ir; 3 Assistant Professor, Department of Biostatistics, Faculty of Nursing and Midwifery. Email: saeed.teshnizi@gmail.com. https://orcid.org/0000-0002-5575-6855 Hormozgan ‎University of Medical Sciences Department of Biostatistics Faculty of Nursing and Midwifery Iran saeed.teshnizi@gmail.com; 4 Assistant Professor, Clinical Research Development Center, Shahid Mohammadi Hospital. Email: nedaa.s.ahmadi@gmail.com. https://orcid.org/0000-0003-1383-5915 Hormozgan ‎University of Medical Sciences Clinical Research Development Center Shahid Mohammadi Hospital Iran nedaa.s.ahmadi@gmail.com; 5 Master's student in medical surgical nursing, student research committee. Email: yasinmoradi489@gmail.com. https://orcid.org/0009-0007-0983-772X Hormozgan ‎University of Medical Sciences student research committee Iran yasinmoradi489@gmail.com; 6 Hormozgan ‎University of Medical Sciences, Bandar Abbas, Iran. University of Hormozgan Hormozgan ‎University of Medical Sciences Bandar Abbas Iran

**Keywords:** Diabetes Mellitus, self care, treatment adherence and compliance, aged, control groups., Diabetes Mellitus, autocuidado, cumplimiento y adherencia al tratamiento, anciano, grupos contro**l.**, Diabetes Mellitus, autocuidado, cooperação e adesão ao tratamento, idoso, grupos controle.

## Abstract

**Objective.:**

To examine the impact of a self-care program designed using Peplau's theory on adherence and self-care in elderly diabetic patients.

**Methods.:**

This semi-experimental study involved 102 elderly diabetic patients from a diabetes clinic in Hormoz, Iran, in 2023. Participants were randomly allocated to either the control group (*n*=51) or the intervention group (*n*=51). Before and two weeks after the intervention, participants completed a demographic information questionnaire, the Modanloo Adherence to Treatment Questionnaire for Patients with Chronic Illness, and the Summary of Diabetes Self-Care Activities Scale. The intervention group received a self-care educational program based on Peplau's therapeutic communication theory, delivered in three phases: orientation, working, and termination. The program focused on key diabetes self-care factors including diet, medication adherence, physical activity, blood sugar monitoring, and foot care. Educational sessions were conducted in small groups or individually in the clinic’s education room. The control group received routine educational content provided by the diabetes clinic.

**Results.:**

The findings showed that the difference between the pre-post mean scores was significantly higher in the intervention group compared with the control group in the total self-care score, as well as in its dimensions: diet, blood sugar regulation, and foot care (*p*<0.001). On the other hand, in terms of adherence, no significant difference was observed in the mean difference between groups for the total score (*p*=0.307), although a statistical difference was found in the dimensions of willingness to participate in treatment (*p*=0.035) and ability to adapt (*p*<0.001).

**Conclusion.:**

The self-care educational program based on Peplau's theory improved the self-care and two dimensions of the adherence: willingness to participate in treatment and ability to adapt in diabetic patients.

## Resumen

**Objetivo.:**

Examinar el impacto de un programa de autocuidado diseñado a partir de la teoría de Peplau sobre la adherencia al tratamiento y el autocuidado en pacientes ancianos diabéticos.

**Métodos.:**

Estudio experimental en el que participaron 102 pacientes diabéticos de edad avanzada de una clínica de diabetes de Hormoz, Irán, en 2023. Los participantes fueron asignados aleatoriamente al grupo de control (*n*=51) o al grupo de intervención (*n*=51). Antes y dos semanas después de la intervención, los participantes completaron un cuestionario de información demográfica, el Cuestionario Modanloo de Adherencia al Tratamiento para Pacientes con Enfermedades Crónicas y la Escala abreviada de Actividades de Autocuidado de la Diabetes. El grupo de intervención recibió un programa educativo de autocuidado basado en la teoría de la comunicación terapéutica de Peplau, impartido en tres fases: orientación, trabajo y finalización. El programa se centró en factores clave del autocuidado de la diabetes, como la dieta, el cumplimiento de la medicación, la actividad física, la monitorización de la glucemia y el cuidado de los pies. Las sesiones educativas se llevaron a cabo en pequeños grupos o individualmente en la sala de educación de la clínica. El grupo de control recibió contenidos educativos rutinarios proporcionados por la clínica de diabetes.

**Resultados.:**

Los resultados mostraron que la diferencia entre las puntuaciones medias pre-post fue significativamente mayor en el grupo de intervención en comparación con el grupo de control en la puntuación total de autocuidado, así como en sus dimensiones: dieta, regulación de la glucemia y cuidado de los pies (*p*<0.001). Por otro lado, en cuanto a la adherencia, no se observaron diferencias significativas entre las medias entre grupos para la puntuación total (*p*=0.307), aunque sí se encontró una diferencia estadística en las dimensiones de disposición a participar en el tratamiento (*p*=0.035) y capacidad de adaptación (*p*<0.001).

**Conclusión.:**

El programa educativo de autocuidado basado en la teoría de Peplau mejoró el autocuidado y dos dimensiones de la adherencia: la voluntad de participar en el tratamiento y la capacidad de adaptación en pacientes diabéticos.

## Resumo

**Objetivo.:**

Examinar o impacto de um programa de autocuidado desenvolvido com base na teoria de Peplau na adesão ao tratamento e no autocuidado em pacientes idosos diabéticos.

**Métodos.:**

Estudo experimental envolvendo 102 pacientes diabéticos idosos de uma clínica de diabetes em Hormoz, Irã, em 2023. Os participantes foram aleatoriamente designados para o grupo de controle (*n*=51) ou o grupo de intervenção (*n*=51). Antes e duas semanas após a intervenção, os participantes preencheram um questionário de informações demográficas, o Questionário Modanloo de Adesão ao Tratamento para Pacientes com Doenças Crônicas e a Escala Abreviada de Atividades de Autocuidado com Diabetes. O grupo de intervenção recebeu um programa educacional de autocuidado baseado na teoria da comunicação terapêutica de Peplau, ministrado em três fases: orientação, trabalho e conclusão. O programa se concentrou em fatores-chave do autocuidado do diabetes, como dieta, adesão à medicação, atividade física, monitoramento da glicemia e cuidados com os pés. As sessões educacionais foram conduzidas em pequenos grupos ou individualmente na sala de educação da clínica. O grupo de controle recebeu conteúdo educacional de rotina fornecido pela clínica de diabetes.

**Resultados.:**

Os resultados mostraram que a diferença entre as médias das pontuações pré-pós foi significativamente maior no grupo intervenção em comparação ao grupo controle no escore total de autocuidado, bem como em suas dimensões: dieta, regulação da glicemia e cuidados com os pés (*p*<0.001). Por outro lado, em relação à adesão, não foram observadas diferenças significativas entre as médias entre os grupos para o escore total (*p*=0.307), embora tenha sido encontrada diferença estatística nas dimensões disposição para participar do tratamento (*p*=0.035) e adaptabilidade (*p*<0.001).

**Conclusão.:**

O programa educacional de autocuidado baseado na teoria de Peplau melhorou o autocuidado e duas dimensões de adesão: disposição para participar do tratamento e adaptabilidade em pacientes diabéticos.

## Introduction

The global population is rapidly aging.^1^Currently, approximately 600 million people worldwide are aged 60 and over, and this number is expected to reach 2 billion by 2050. According to projections by the United Nations, it is anticipated that by 2025, 10.5% of the population in Iran will be over 60 years old, and this figure is expected to increase to 21.7% by 2050.^2^Although aging itself is not considered a disease, the physiological changes associated with aging increase the likelihood of developing diseases. In the elderly, the epidemiological pattern of diseases has shifted towards a higher prevalence of chronic diseases.^3^Diabetes is one of the common chronic diseases among the elderly, requiring special attention and management.^4^A study with over 1.3 million participants found that 98% of adults with type 2 diabetes have at least one accompanying chronic condition, and nearly 90% have at least two.^5^ Additionally, a study in Iran revealed that the prevalence of diabetes among individuals over 60 years old was 29.03%.^6^This disease can lead to serious problems and complications, including an increased risk of cardiovascular diseases, kidney damage, and vision problems in the elderly. People with diabetes may have poor self-care behaviors; therefore, identifying barriers to self-care is also a crucial step in improving or enhancing self-care behaviors.^7^Self-care is an active and practical process managed by the patient, aiming to monitor medication treatment and use medications appropriately, follow a healthy diet, exercise, care for the feet, prevent diabetic wounds, and control blood glucose levels.^8^Studies have shown that self-care behaviors in diabetic patients are at a low level, and individuals with less ability to care for themselves are at greater risk of developing diabetic complications. In elderly patients with diabetes, neglecting self-care leads to poor treatment adherence and an increased risk of serious complications.^9^

Non-adherence to treatment regimens in diabetic patients is associated with frequent hospitalizations, failure to receive therapeutic benefits, high treatment costs, and a large number of physician visits. The mortality rate in patients who do not adhere to their treatments is twice as high as in those who do.^10^According to the World Health Organization, adherence refers to the extent to which an individual's behavior-such as taking medication, following a diet, or implementing lifestyle changes-corresponds with the recommendations provided by healthcare personnel.^11^Some studies have shown that 4 to 31 percent of diabetic patients never proceed to obtain the prescribed medications, and some refrain from using them after obtaining their prescriptions to the extent that 30 to 50 percent of diabetic patients refrain from using blood pressure and lipid-lowering medications, which play an important role in reducing cardiovascular events, during the first three months of drug therapy.^12^In this regard, nurses play an important role in encouraging patients to participate in the self-care process and adhere to treatment.^13^

Nrsing theories are considered essential for guiding nurses to advance the nursing profession and provide standard care. From Peplau's nursing theory perspective, nurses have various roles, including educator, counselor, patient advocate, facilitator, and source of information, all of which require appropriate patient communication for proper execution, based on the circumstances.^14^Peplau's theory emphasizes the importance of interpersonal communication between the nurse and patient as a central component of effective care. According to Peplau, interpersonal communication occurs in phases: orientation, identification, exploitation, and resolution. These phases allow nurses to establish a relationship with the patient, understand their needs, provide education, and guide them through their treatment process. By utilizing these communication techniques, nurses can help patients navigate their concerns, reduce anxiety, and encourage active participation in their care.^15^In the context of diabetes management, effective communication is crucial in motivating patients to adopt self-care behaviors and adhere to treatment regimens. Interpersonal communication allows nurses to understand the individual needs and concerns of elderly diabetic patients, tailoring interventions that address barriers to self-care and treatment adherence.

Since Peplau's theory introduces a clear framework for effective communication during nursing care, it can be used to provide patients with the most effective education and involve them actively in the education process.^16^ Considering the limited number of diabetes centers and associations in Iran and the difficulty in accessing them for the elderly living in rural areas, self-care education and self-care levels in these patients are low. Currently, patients receive necessary training through educational pamphlets and face-to-face interactions in a short period, with insufficient attention to their educational needs, expectations, knowledge levels, and understanding. Disorders in self-care and treatment adherence can directly affect the quality of life of elderly diabetic patients. Therefore, this research was designed to determine the impact of a self-care educational program based on Peplau's theory on treatment adherence and self-care in elderly diabetic patients.

## Methods

**Figure 1:**
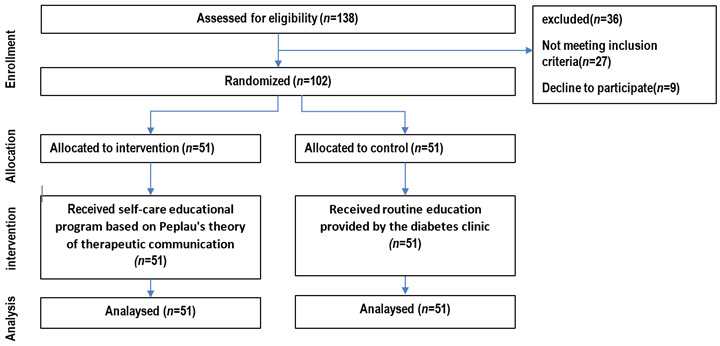
Flow diagram of the progress through the phases of a parallel randomised trial of two groups

Study Design. This was a semi-experimental, parallel-group study conducted at the Diabetes Specialty Clinic in Hormoz, Iran, in 2023. (Figure 1). The study was designed to evaluate the effect of a self-care educational program based on Peplau’s theory of therapeutic communication on adherence to treatment and self-care among elderly diabetic patients. Ethical approval was obtained from the relevant institutional review board, and all participants provided informed consent before enrollment.

Participants. The study included elderly patients aged 65 years or older with a confirmed diagnosis of diabetes by an internal medicine specialist, residing in Bandar Abbas, and undergoing treatment with either oral medication or insulin for at least one year. Participants were required to demonstrate the ability to perform self-care activities independently and have no significant auditory or visual impairments. Exclusion criteria included withdrawal from the study, deterioration in health (e.g., impaired consciousness or death), or any change in condition that affected the ability to communicate.

Sample Size. Sample size calculations were based on mean self-care scores reported by Markel-Reed et al. (2018). Using a confidence level of 95% (Z = 1.96), a power of 80% (Z = 0.84), and expected means and standard deviations (μ1 = 42.83, μ2 = 37.86; S1 = 8.52, S2 = 11.33), a sample size of 51 participants per group was determined. Calculations were performed using WinPepi software (version 11.65).

Randomization. Participants were allocated to either the intervention or control group using block randomization with 17 six-unit blocks generated by Randomization Main software. This ensured equal distribution across groups.

Interventions. The control group received routine education provided by the diabetes clinic. The intervention group participated in a self-care educational program based on Peplau's theory of therapeutic communication, delivered in three phases: orientation, working, and termination. Educational sessions were held face-to-face in small groups (maximum of two participants) or individually in the clinic’s education room. The phases of the intervention were: (i) *Orientation:* Two sessions (20-30 minutes each) were conducted over two weeks. The first session focused on rapport-building and explaining the study purpose, while the second addressed patients' strengths and challenges; (ii) *Working:* Two sessions (20-30 minutes each) were held one week apart, covering topics such as diet, medication adherence, physical activity, blood sugar measurement, and foot care using educational brochures and video clips; (iii) *Termination Phase:* Two sessions (20-30 minutes each) were held one week apart. In the first session, participants' questions were addressed, and the second session facilitated group discussions to share experiences and reinforce learning. Educational content was derived from the "Healthy Lifestyle Volume 3" handbook by the Ministry of Health, tailored to elderly patients. To accommodate participants' needs, session schedules were coordinated via telephone, and companions were allowed to attend.

Outcomes. The primary outcomes were self-care ability and adherence to treatment. These were assessed using: (i) *Modanloo Adherence to Treatment Questionnaire:*^17^ A 40-item validated tool measuring adherence across seven domains. Scores were categorized as very good (75-100%), good (50-74%), average (26-49%), and weak (0-25%); (ii) *Summary of Diabetes Self-Care Activities (SDSCA) Scale:*^18^ A 15-item scale assessing self-care behaviors, with scores categorized as good (76-100), moderate (51-75), and poor (≤50). Reliability was confirmed via Cronbach’s alpha for adherence (α = 0.736) and self-care (α = 0.811).

Data Collection and Analysis. Baseline demographic and clinical data were collected via structured interviews. Questionnaires were administered pre- and post-intervention by a researcher blinded to group allocation. Post-tests were conducted two months after the intervention. Data analysis was performed using SPSS (version 26.0): (a) Between-group differences were analyzed using independent t-tests or Mann-Whitney U tests (for non-normal distributions); (b) Within-group differences were assessed using paired t-tests or Wilcoxon tests; (c) Relationships between demographic/clinical variables and outcome changes were explored using Pearson correlation, ANOVA, and independent t-tests. A p-value of <0.05 was considered statistically significant for all analyses.

Ethical Issues. This study was conducted in accordance with ethical principles outlined in the Declaration of Helsinki. Ethical approval was obtained from the Iran National Committee for Ethics in Biomedical Research (Approval Number: IR.HUMS.REC.1402.033). Written informed consent was obtained from all participants prior to their enrollment, ensuring voluntary participation and understanding of the study's purpose and procedures. The confidentiality and anonymity of participants were strictly maintained, with all data being de-identified and securely stored. Participants were informed of their right to withdraw from the study at any time without any consequences. Additionally, the educational intervention posed no harm and was designed in alignment with standard care practices. The study adhered to the ethical guidelines set by the Iran National Committee for Ethics in Biomedical Research, and all potential conflicts of interest were transparently disclosed.

## Results

The results of comparing the frequency distribution of demographic characteristics of the two intervention and control groups along with the results of the chi-square test in Table 1 indicated that the frequency distribution of variables in the two intervention and control groups did not have a statistically significant difference (*p* > 0.05).

**Table 1:** Demographic variables of patients with type 2 diabetes divided into two study groups

***p*-value**	Control (*n*=51)	Intervention (*n*=51)	Categories	Variables
*n* (%)	*n* (%)
0.547	31 (39.22)	28 (45.1)	Male	Gender
	20 (60.78)	23 (54.9)	Female	
0.501	12 (23.53)	15 (29.41)	Single	Marital status
	39 (76.47)	36 (70.59)	Married	
0.375	12 (76.47)	16 (68.63)	1 to 2 years	Duration of diabetes
	39 (23.53)	35 (31.37)	More than 2 years	
0.445	43 (15.69)	40 (21.57)	Yes	Medical history
	8 (84.31)	11 (78.43)	No	
0.676	28 (54.9)	29 (56.86)	Illiterate	Level of education
	9 (17.65)	10 (19.61)	Primary	
	14 (27.45)	12 (23.53)	diploma	
0.542	21 (41.18)	20 (39.22)	Employee	Employment status
	6 (11.76)	10 (19.61)	Unemployed	
	24 (47.06)	21 (41.18)	Retired	
0.208	37 (72.55)	31 (60.78)	City	Place of residence
	14 (27.45)	20 (39.22)	Village	
0.912	10 (19.61)	10 (19.61)	Alone	Family composition
	28 (54.9)	25 (49.02)	With spouse	
	11 (21.57)	14 (27.45)	With spouse and children	
	2 (3.92)	2 (3.92)	Other	
0.135	44 (86.27)	38 (74.51)	Yes	Family history of diabetes
	7 (13.73)	13 (25.49)	No	
0.290	14 (27.45)	19 (37.25)	Yes	Tobacco use
	37 (72.55)	32 (62.75)	No	
0.510	37 (72.55)	33 (64.71)	None	Tobacco type
	8 (15.69)	5 (9.8)	Cigarettes	
	0 (0)	7 (13.73)	Pip	
	6 (11.76)	6 (11.76)	Hookah	
0.346	6.65±70.65	3.93±69.55	Age (mean±SD)	

As depicted in Table 2, significant improvements in the dietary regimen (*p*<0.001) physical activity (*p*<0.001), blood sugar regulation (*p*<0.001), regular medication intake (p=0.047), and foot care (*p*<0.001) compared to before the intervention was observed. However, no significant differences were observed in smoking (*p*=1.0) and physical activity (*p*=0.192) dimensions between the average changes before and after the intervention in the two groups (*p*>0.05). Additionally, independent t-test results indicated a significantly higher mean self-care score among elderly individuals with diabetes after the intervention compared to before the intervention (*p*<0.001). Moreover, the mean changes (increases) in the total self-care score for the intervention group were significantly higher than the control group (4.3 vs 0.02; *p*<0.001). Based on questionnaire scoring, the level of self-care of the study participants was evaluated as average before and after the intervention (self-care score less than or equal to 50).

**Table 2:** Comparison of mean self-care and its dimensions before and after the intervention phase by groups

Pre-and post-comparison test		Difference before and after	After intervention	Before intervention	Groups	Variables
***p*-value**	Statistics					
0.001>†	-4.80	6.33+8.26	24.94±3.72	18.61±7.81	Intervention	Diet
†0.16	-1.41	0.2+0.98	19.31±7.94	19.12±8.11	Control	
		0.001>	0.001>	0.772	^Independent two-group comparison test*^	
0.001>‡	1.00	0.0+0.2	2.69±2.16	2.69±2.15	Intervention	Physical activity
0.13‡	-1.51	-0.11+0.46	2.75±2.08	2.84±2.19	Control	
		0.192	0.834	0.772	^Independent two-group comparison test*^	
0.001>‡	-5.87	3.1+2.08	6.25±2.31	3.16±2.49	Intervention	Blood sugar regulation
0.16‡	-1.41	0.04+0.2	3.12±2.33	3.08±2.34	Control	
		0.001>	0.001>	0.911	^Independent two-group comparison test*^	
0.180‡	-1.34	0.06+0.31	6.41±1.72	6.35±1.88	Intervention	Regular use of medication
0.16‡	-1.41	-0.04+0.2	6.51±1.67	6.55±1.59	Control	
		0.047	0.978	0.743	^Independent two-group comparison test*^	
0.001>‡	-5.72	3.47+2.56	7.98±4.99	4.51±5.34	Intervention	Foot care
0.18‡	-1.34	0.06+0.31	4.61±5.63	4.55±5.66	Control	
		0.001>	0.001>	0.786	^Independent two-group comparison test*^	
1.000‡	0.00	0.00	0.88±0.33	0.88±0.32	Intervention	Smoking
1.000‡	0.00	0.00	0.86±0.34	0.86±0.34	Control	
		00.1	0.768	0.768	^Independent two-group comparison test*^	
0.001>	7.47	7.73+7.39	49.1±7.71	41.37±8.24	Intervention	Self-care (total)
†0.811	0.240	0.02+0.58	37.16±11.40	37.14±11.30	Control	
		0.001>	0.033	0.204	^Independent two-group comparison test^^	

†Paired t-test, ‡Wilcoxon test, ^Independent t-test, *Mann-Whitney U test

The results showed that the difference between the pre-post mean scores was significantly higher in the intervention group compared with the control group in the total self-care score, as well as in its dimensions: diet, blood sugar regulation, and foot care (p<0.001). On the other hand, in terms of adherence, no significant difference was observed in the mean difference for the total score (p=0.307), although a statistical difference was found in the dimensions of willingness to participate in treatment (p=0.035) and ability to adapt (p<0.001).

The results of the independent t-test, depicted in Table 3, showed that the mean total score adherence to treatment in the elderly diabetic intervention group, before intervention and after intervention, did not have a statistically significant difference (p=0.58). Also, the Wilcoxon test results showed no statistically significant difference in the total questionnaire score difference between the two groups (p=0.58, -0.43 vs 0.06). For dimensions of willingness to participate in treatment (p=0.035), doubt in treatment implementation (p=0.012), and ability to adapt (p=0.001) in the current study, willingness to participate in treatment, doubt in treatment implementation, and ability to adapt after intervention in the intervention group were higher than the control group. No other dimensions showed a significant difference between the average changes in both groups before and after the intervention (p>0.05). Considering the scoring of adherence to the treatment questionnaire in this study, individuals who scored more than 50% (66% of individuals) were classified as individuals with a good level of treatment adherence. 

**Table 3:** Comparison of mean adherence to treatment and its dimensions before and after intervention separately in two groups

Pre-and post-comparison test		Difference before and after	After intervention	Before intervention	Groups	Variables
***p*-value**	Statistics					
0.67‡	-0.43	0.04+2.78	22.41±2.29	22.37±2.32	Intervention	Interest in treatment
0.08‡	-1.73	0.06+0.24	20.35±2.7	20.29±2.73	Control	
		0.70	0.001>	0.001>	^Independent two-group comparison test*^	
0.06‡	-1.91	0.18+0.65	22.41±2.29	22.24±2.28	Intervention	Willingness to participate in treatment
0.16‡	-1.41	0.04+0.2	20.33±2.72	20.29±2.73	Control	
		0.350	0.001>	0.001>	^Independent two-group comparison test*^	
0.001>‡	-2.63	0.79+3.17	15.95±2.7	15.16±2.03	Intervention	Ability to adapt
0.10‡	-1.63	-0.1+0.41	14.84±3.02	14.94±2.99	Control	
		0.001>	0.001>	0.566	^Independent two-group comparison test*^	
0.32‡	-1.00	0.02+0.14	25±0	24.98±0.14	Intervention	Integration of treatment with life
0.07‡	-1.84	-0.14+0.53	24.71±0.92	24.84±0.78	Control	
		0.166	0.048	0.515	^Independent two-group comparison test*^	
0.06‡	-1.89	0.1+0.94-	14.25±4.17	14.35±4.06	Intervention	Adherence to treatment
0.33‡	-0.98	0.1+0.36	14.61±4.15	14.51±4.34	Control	
		0.316	0.765	0.681	^Independent two-group comparison test*^	
0.13‡	-1.51	0.12+0.55	20.27±2.67	20.16±2.72	Intervention	Commitment to treatment
0.18‡	-1.34	0.06+0.31	21.04±2.25	20.98±2.29	Control	
		0.058	0.011	0.04	^Independent two-group comparison test*^	
0.17‡	-1.37	0.18+0.84	12.69±1.82	12.51±1.73	Intervention	Doubts in treatment implementation
0.10‡	-1.63	0.08+0.34	12.49±1.87	12.41±1.88	Control	
		0.012	0.777	0.515	^Independent two-group comparison test*^	
0.58†	0.28-	0.43±9.14	132.2±7.7	131.77±7.46	Intervention	Adherence to treatment (total)
0.69†	-0.39	0.06+1.28	129.24±8.06	129.18±8.15	Control	
		0.307	0.023	0.057	^Independent two-group comparison test^^	

†Paired t-test, ‡Wilcoxon test, ^Independent t-test, *U-Man-Whitney test

## Discussion

The present study was conducted to determine the impact of implementing a self-care educational program based on Peplau's theory on self-care and treatment adherence of elderly patients with diabetes. The results of the current research indicated that the difference in average scores of self-care before and after the intervention in the intervention group patients at the end of the intervention was significantly higher than the difference in average scores of self-care of the control group patients. In other words, implementing a self-care educational program based on Peplau's theory had a significant impact on self-care in elderly patients with diabetes. In line with these results, Fernandes et al.^19^ showed that implementing a self-care educational program based on Peplau's theory improved the level of self-care in patients with type 2 diabetes, and the use of this theory in the self-care of chronic patients, including type 2 diabetes, had a positive impact, which is consistent with our findings.^19^In the present study, the intervention group significantly adhered more to dietary recommendations and foot care than the control group. Khiyali *et al*.,^20^ and Hoshamandja *et al.*,^21^in Iran, Hiloo *et al.* in Ethiopia^22^, and Lee *et al.*,^23^in Korea also found similar results in their studies. Although differences in teaching methods and measurement tools used in these studies make it difficult to compare them, they collectively show a positive direction about the impact of educational interventions on adherence to diabetic nutrition and foot care. In terms of the sub-scale of regular drug usage, there was no significant difference in the average score before and after the intervention in the intervention group. The findings of the present research are consistent with the results of studies by Khayali *et al.*,^20^ and Hooshmandja *et al.*^21^

The reason for the significant increase in patient drug adherence in the mentioned studies may be justified by reminding individuals through mobile phone follow-up in addition to emphasizing regular drug use to prevent serious diabetes complications. However, in the present study, education on regular drug use was only in one face-to-face session, which may be challenging for elderly individuals to remember. On the blood sugar control subscale, a significant difference was observed between the average score difference before and after the intervention in both groups, which is consistent with previous studies on diabetic patients.^20^^,^^21^ In most developing countries, there is a significant gap between practical recommendations and provided care, which leads to poor blood sugar control.^24^Despite differences in educational methods and measurement tools used in these studies, overall, the results show that educational interventions can have a positive impact on controlling blood sugar in patients with type 2 diabetes. In the present study, no significant impact on physical activity was observed, which is not consistent with the results of study by Shojaeezadeh *et al*.,^25^ but is in line with the study by Hailu *et al.*^22^In this research, given the advanced age of the study participants, simple and practical education including walking for about ten minutes after meals and avoiding sitting for more than an hour did not create the necessary motivation to improve physical activity later. On the smoking subscale, no significant difference was observed between the two groups. *Hailu et al.*^22^ also did not report a significant difference in smoking in their studies. It seems that stronger motivations are needed for changing habits like smoking, and education alone may not create strong motivation in individuals.

The present research findings indicate that the average adherence to treatment after intervention in the intervention and control groups does not have a statistically significant difference. Therefore, it can be concluded that the educational intervention based on Peplau’s theory could not have a significant effect on the overall adherence to treatment score of elderly diabetic patients. The educational intervention may provide to patients, due to being short or inadequate in meeting their real needs, may not have the ability to bring about significant changes in treatment adherence. Furthermore, environmental factors such as family support or cultural and social constraints can influence the effectiveness of the educational intervention Most of the search results highlight positive applications or outcomes of Peplau's theory in various nursing and healthcare contexts. A study on hospitalized older adults in cardiac intensive care units found that using Peplau's communication theory increased patient satisfaction with nursing care compared to a control group.^26^ In addition, A study on elderly people with diabetes mellitus found that effective interpersonal relationships in nursing care correlated positively with greater treatment adherence to specific dietary recommendations.^27^Although this study did not explicitly use Peplau's theory, it highlights the importance of nurse-patient relationships in diabetes self-care.

The results showed that the self-care ability of the participants in this study was weak, which was consistent with the results of the study by Borhaninejad *et al.*^28^. However, in the studies by Robbat Sarposhi *et al.*,^29^the level of self-care was assessed as average, which was inconsistent with the results of the present study. Discrepancies in the results of studies may be due to differences in measurement tools and population characteristics. Additionally, the timing and location of the studies may also play a role in these differences. The results of the present study indicated good adherence to treatment among the elderly participants. However, the study by Tanharo *et al.*^30^on diabetic patients showed poor treatment adherence, which was inconsistent with the present study. The difference in these results could be attributed to the limited sample size of the present study and the differences in the age groups of the study participants. In the present study, there was no correlation between age and treatment adherence, possibly because the study participants were elderly individuals over the age of 65. Tanharo *et al.*^30^ showed that with increasing age, treatment adherence also increased, indicating a higher risk of diabetes complications with age. 

Conclusion. Implementing a self-care educational program based on Peplau's theory has a positive impact on self-care in elderly diabetic patients. However, according to the results, the mentioned educational program did not have a positive impact on treatment adherence in these patients. Therefore, the nurse's role as healthcare providers for patients can be effective in reducing complications of chronic diseases, including diabetes.
